# Topics of Public Interest

**Published:** 1904-02

**Authors:** 


### TOPICS OF PUBLIC INTEREST.

### THE OPTOMETRY BILL ONCE MORE.

ITS PERNICIOUS FEATURES—PHYSICIANS SHOULD SUPPORT THE COMMITTEE IN EFFORTS TO DEFEAT IT—A STIRRING APPEAL.

MEDICAL SOCIETY OF THE STATE OF NEW YORK,
OFFICE OF THE COMMITTEE ON LEGISLATION.

*To the Medical Profession of the State of New York:*

An optical society, composed of so-called "refracting opticians," has issued a circular letter to the medical profession, asking for the indorsement of a bill about to be introduced in the legislature, legalising the "practice of optometry."

This measure is not a new thing; we have met it several times in the past, and it has only been necessary to expose its pernicious features to defeat it. It will be necessary to do this again and again, until the public awakens to the fact that measures such as this are only the efforts of incompetent people to evade the medical laws, in order that they may prey upon the community without fear of molestation, and then suitable provisions will be made to suppress them.

It is to be hoped that members of the medical profession will not be influenced by the specious arguments of these opticians to give them any encouragement or indorsement; for any success they may attain will be the signal for a host of osteopaths and other similar sects to ask for similar privileges. There are no good reasons why opticians should be granted any special privileges. They undertake to do a work which they are not in any way qualified to perform, and there is no doubt, in the minds of those who are competent to judge, that they do incalculable harm and injury. They, in common with all others who treat physical defects and infirmities, should have a medical education before they are allowed to pose before the public as competent to do the work they pretend to be able to do. It cannot be expected that future physicians will be willing to undertake a course of studies to prepare themselves for the work of caring for the sick and physically defective, if, after having fulfilled all the legal requirements, they find themselves obliged to compete with others who have received almost identical rights and privileges with practically no preparation. If medical laws are necessary to the proper protection of the community, they should apply equally to all who make a business of advising or treating people, regardless of the disease or defect from which they suffer, or of the methods or measures employed. It is impossible for the state to discriminate between physicians, opticians, and the various "pathies" without injury to the cause of professional elevation. A single standard must be created for all, and with its requirements all should comply.

The committee on legislation of the Medical Society of the State of New York has fought these legislative battles successfully in the past and will continue to do so if the profession will support it as heretofore it always has done. The members of the legislature have no knowledge of the merits of this and similar measures, and look to the medical profession for advice and guidance. The legislature is made up of fair-minded, intelligent, and loyal citizens; and when pernicious laws affecting the public health are enacted, it is, as a rule, because of the neglect of citizens who are not members of the legislature to do their duty. We urge the members of the medical profession resolutely to oppose this "optometry bill." If it does so the defeat of the bill is assured.

(Signed) FRANK VAN FLEET,

Chairman, Committee on Legislation,
Medical Society of the State of New York.

### A PLEA FOR UNIFICATION AND UNIFORM ORGANISATION.

By J. N. McCORMACK, M. D., Bowling Green, Ky.
Chairman of the Committee on Organisation of the American Medical Association.

*To the Medical Profession of the State of New York:*

After correspondence on the subject with members of the joint conference committee, and others, more especially on the society side, I have been requested, as chairman of the committee which has had this work in charge from its inception, to prepare for publication an explanation of the plan of organisation for state and county societies which has been so generally adopted in the last two or three years. As the plan was prepared and is recommended for universal adoption, is already in satisfactory operation in 32 states and in over 1,500 counties, and is likely to be adopted in nearly all of the other states as soon as their annual meetings occur, as the subject is one of interest at this time to every member and well wisher of our profession, and, more especially, as the preparation of the plan for general adoption, makes my committee as responsible to you for anything wrong in it as though we were members of your county and state societies, it seems entirely proper that the request should be complied with, and an attempt will be made to place beyond question the truth of the following propositions:

1. That existing professional conditions and estrangements are useless and senseless, that they are hurtful almost beyond calculation to both the profession and the public welfare, and that they are remediable.

2. That the plan of organisation is based upon our system of civil government, that it was prepared with much care and unselfishness, and that it contains nothing untried, experimental or impracticable.

3. That the county society is not only the unit of the organisation, but the source of all authority and power in both the state and national bodies through a representative delegate system, and it is also the only portal of entry to the entire society system.

4. That coincident membership has been an old and perfectly satisfactory method in many states, is essential to complete organisation, and that it is just and right. It would be as unfair to permit a physician to become a member of his county society and enjoy all the benefits arising from the labors of the state society, as to permit one to become a citizen of Ithaca or Troy, without becoming a citizen of the state of New York and bearing the burdens and responsibilities of the same.

5. That amendment of any part of the plan is purposely made easy and is controlled by delegates from, and responsible alone to, the county societies.

6. That the time has come for unification, and that, while union under a defective plan, which the joint body could modify and improve for the common good, would be far preferable to a continuation of existing conditions, the one now proposed has been found excellent and practical after full trial in other states, and, having been prepared away from New York contentions and controversies, it offers a basis for compromise honorable alike to both factions and to neutrals.

In order to establish these propositions clearly and satisfactorily, a brief history of this reorganisation movement is necessary. From the very outset the magnitude of our task grew upon us. Although in name medical societies—city, district, state and national—existed without number, and medical machinery and high-sounding official titles were provided sufficient for the civilised world, membership in most of them was small and little more than nominal, and, outside of a few states and localities, the profession was so torn with dissensions that it was in chronic disgrace in its own and the public estimation, and in a large measure robbed of its power, by legislation or otherwise, to help either itself or the public, for whose benefit it was supposed to exist. Into a profession already so overcrowded that there was less room and remuneration for the average man than for clerks and skilled laborers, commercial medical colleges were annually turning loose thousands of new graduates, many poorly equipped for intelligent modern practice, and all of them without an hour's training in professional conduct, business methods, or the importance of society membership, in which all should have been drilled with the care due to subjects to become of so much importance as soon as they come to serve the public, and face the problem of earning an honorable livelihood.

As a result, largely, of such lack of training, we found that of the 120,000 registered physicians in this country, only about 30,000, one in four, had ever been members of any kind of society, local, state or national. Most cities were little better organised than the country districts, a large submerged element being often found within an easy walk of imposing medical and post-graduate schools, libraries and hospitals, who had long lost hope of advancement, looked upon their profession only as a doubtful means of support, while those in the higher ranks wrangled and slandered one another, and brought odium on all, and christian science and other forms of bald, naked quackery, took the cream of a patronage which a united, self-respecting and honored profession would have deserved and received.

It was soon found, however, that these conditions, deplorable alike to the profession and public, were not universal. In Alabama, for instance, under a model plan of organisation there resulted a united profession, with over 1,500 of the 1,700 physicians members of the county and state societies. Medical opinion was sought for the guidance of law-makers, executives and courts, the profession was honored and independent, and quackery was unknown. Kentucky, striving for the same ends under less perfect methods, had had no itinerant or advertising doctor within her borders for ten years, and the profession was aspiring to its rightful place as a directing force in public affairs. In a dozen cities also, usually of the lesser rank in population, under similar influences, harmony, coöperation, and measureable prosperity prevailed, and was increasing.

Inquiry soon demonstrated that the personnel of the profession was much the same everywhere, and the difference in the conditions found were apparently entirely due to difference in methods of organisation. After all proper allowance for personal equation in rulers, governments are liberal or despotic as determined by their organic laws, and, probably quite as much as with a nation, the constitution and by-laws of a medical society will set the compass and shape the policy which will largely determine the future for both itself and its individual members.

Influenced by such belief and considerations, my committee began its work four years ago by grouping these provisions for state and local societies which had resulted in the most effective organisations, and with great care made our first draft of a constitution and by-laws for submission to, and discussion by, conferences of leading men in the various medical centers where there was a reasonable degree of harmony. Later the improved draft was published in the medical press and in pamphlet form and given the widest possible publicity. As we were performing an important, unselfish and gratuitous public service for the common good, advice and frank criticism were cordially asked from all, and these came freely and helpfully, especially from the better organised states and cities.

With such assistance, and in the light of such criticism, keeping always in mind that the plan should be democratic and conform to our methods of civil government, with local self-government by the county societies as the central idea, a simple but comprehensive system was gradually evolved, almost ideal in its outline and possibilities, which, after full trial under the most diverse professional conditions, has proven entirely practical. It is so flexible that it can be as easily adapted to the Cook County Medical Society at Chicago, with 1,800 members, as to the sparsely settled counties in North Carolina or California with ten members. Under its operations in one year in Michigan the membership increased from 560 to 2,100; in Kentucky, from 390 to 1,600; in Tennessee, from 300 to over 1,000; in Missouri, from 400 to 1,500; in Arkansas, from 190 to 703, and in like proportion in every other state adopting it.

It is a thoroughly democratic and American plan, using these much abused terms in their broadest and best sense. As has been said, the county society is made the broad foundation and source of authority for everything, as well as the portal of entry to both the state and national bodies. If a physician stands well enough at home to secure membership in his county society, he is eligible for membership and for any position of honor or trust in the state and national bodies, and, if he loses membership at home, subject to the right of appeal, he loses out everywhere.

The house of delegates, the legislative and business body of the state society, like the general assembly of a state, is composed of representatives from the county and district societies, and such delegates are freed from political influence so far as possible by making them ineligible for any of the offices within their gift, and they are responsible alone to the constituencies electing them. As they are in session but two or three days, in order to make the transaction of any business possible, unlike senators and representatives, they sit as one body. This gives a body small enough for deliberative work, and yet gives county societies remote from the place of meeting, and all others, an equal and only an equal, voice in all business affairs, if only the delegates are in attendance.

Carrying out the same principle of local self-government, this house of delegates, made up directly from county and district societies, elects from the general membership the delegates who, in turn, compose the legislative body of the American Medical Association. As in the house of delegates of state societies, this being a small body, with its members carefully selected and made ineligible for any other position, with the terms of half the members expiring each year, so as always to have men of experience in it, and which can remain in session, if necessary, after the general meeting adjourns, it was hoped to provide a body in which the interest of the profession and of scientific medicine might be considered and promoted in the broadest way.

The question of coincident membership in county and state societies is one which should require little explanation in New York. It has given satisfaction in the association, and has, of course, been under the observation of the society people. You have also had ample opportunity to observe the results of it in the neighboring states of New Jersey and Connecticut, where it has been in operation for years. It has been long the practice in Alabama, with the result that about 90 per cent. of the profession are permanent members, as well as in Massachusetts, Pennsylvania and Indiana, and recently in a total of thirty-two states. It is only one feature of the work, but it is so important that any complete or systematic organisation of the profession is impossible without it. Besides, the justice of it is beyond question. It would be as obviously unfair to permit one to join his county society and enjoy all the benefits of the state society, arising from its legislative, educational, and other expensive and responsible labors, without assisting in its support, as it would be to permit a man, if one could be found so foolish and selfish as to ask it, to become a citizen of Buffalo, Syracuse or Rochester, and enjoy all the benefits and protection of the state government, without becoming a citizen of New York.

It is true that in nearly every state where this plan of organisation has been proposed, as was natural and to be expected, academic and purely theoretical objections have been made to some feature of it, usually to the council, the house of delegates, or to coincident membership. Such objection has always been made in advance of a trial of them, and they have been confined almost entirely to states which have been practically without organisation heretofore. Conditions in Iowa illustrate this point well. The county society at Dubuque, which has reorganised and applied for a charter within the last ten days, raised many such theoretical objections to the plan before they had tried it, while 88 counties in the same state, organised under it, and realising its practical benefits, are loud in praise of the very provisions to which their worthy neighbor at first objected. It is a source of much encouragement that this has been the experience of over 1,500 counties which have reorganised.

It would be pleasant to comment on each provision of the plan in detail if space permitted. Suffice to say that it was intended only as the foundation and frame work for a broad and comprehensive system to be recommended for universal adoption, that nothing was included except for what appeared the best of reasons, and that it is susceptible of infinite elaboration and improvement in detail as the profession of any state or county develops under the catholic and coöperative spirit which it inculcates, and which is so much to be desired.

Attention is called to the fact also, that much contained in the by-laws, where a wide departure is made from the brevity and compactness of expression adhered to in the constitution, is suggestive and educational in character, much of which would not be necessary if such an organisation as is contemplated was already in existence. We thought it as important, under existing conditions of discord and demoralisation, to furnish incentives to this work, to give practical and detailed information as to how it was to be done, and to put the reasons for it all within easy reach of those whose duty it would be to discuss the subject and do the work as to furnish the framework of which, as the result of years of devoted labor, they were to make a complete edifice occupied by a united and harmonious, or at least a decently coöperative and self-respecting profession. For many reasons, too, amendment to the plan is purposely made easy. If after trial in any state anything is found obsolete or impracticable, it can be easily eliminated or replaced by something better adapted to existing needs, by the house of delegates, subject to instruction from the county societies, at any regular meeting, after such amendment has lain upon the table for one day.

Unification in New York has been so long the dream and hope of its own profession, as well as of the entire country, that now, when this seems about to be realised, it may be that a majority of you will consider mere methods and details of organisation important only in so far as they are likely to hasten or delay the union. In part, this view of the matter is both natural and correct. A cordial union under a defective plan, which could be modified and improved to meet the needs of the joint body, when there would be unity of purpose and interest for the common good, would certainly be far better than to perpetuate the two bodies, even with model organisations for both, with the strife, discord and loss of public respect inseparable from such conditions anywhere.

Still, if it can be shown, as the writer believes will appear upon the most critical investigation of what is now proposed, that it furnishes ready to your hands a plan of organisation to which you may build and add with confidence in the results, and which will at the same time hasten unification by furnishing a basis of compromise between factions so long divided that suspicion and jealousy have become so much mental habits that neither will be willing to accept what the other proposes, it should, and I am sure will, receive respectful consideration at your hands.

In the era of good feeling and enthusiasm which must follow unification, under such a broad, liberal and comprehensive plan, which would give an equal voice and power to the individual doctor in every county, organisation should and would make rapid strides, and the possibilities opened up to your profession, and then to your people, would be almost beyond conception. In the face of such possibilities, standing upon the threshold of such accomplishments, petty prejudices and personal interests appear so insignificant that they should find no place in large minds.

Then, as New York would be in perfect alignment with her sister states, with proper education and effort, everything desirable would, in time, be brought within reach of the united profession of the entire union. The complex and difficult problems before the profession of this country incident to our unprecedented advance in population and civilisation, and which must be solved by it, if they are to be solved, will tax its highest intelligence and energies at its best. Provision can then be made for continuous scientific research, and for systematic collective investigation into the causes and prevention of diseases, upon the large, generous lines demanded by the vast interests involved. The vexed problem of medical education can then be taken up with confidence and justly and wisely solved. Reciprocity in licensure and membership between states can then be discussed from the standpoint of the common good and settled upon some equitable basis. Constructive statesmanship can be substituted for the narrow, time-serving political methods of the present in municipal, state and national public health affairs, and our great profession, great even now in spite of its divisions, united, elevated and ennobled, would come to occupy its rightful place as one of the greatest of modern forces for the guidance and protection of our people. It would be an honor, even to New York, to lead in a reform promising such results, and to set an example in complete organisation, which less favored and weaker states would imitate with both pride and profit. First of all, leading to all and above all, in the words of your victorious commander, greater in magnanimity even than in war, "Let us have peace."

